# Filler-Modified Castor Oil-Based Polyurethane Foam for the Removal of Aqueous Heavy Metals Detected Using Laser-Induced Breakdown Spectroscopy (LIBS) Technique

**DOI:** 10.3390/polym12040903

**Published:** 2020-04-13

**Authors:** M. Iqhrammullah, Marlina, R. Hedwig, I. Karnadi, K. H. Kurniawan, N. G. Olaiya, M. K. Mohamad Haafiz, H. P. S. Abdul Khalil, S. N. Abdulmadjid

**Affiliations:** 1 Graduate School of Mathematics and Applied Sciences, Universitas Syiah Kuala, Banda Aceh 23111, Indonesia; m.iqhram@oia.unsyiah.ac.id; 2 Department of Chemistry, Faculty of Mathematics and Natural Sciences, Universitas Syiah Kuala, Banda Aceh 23111, Indonesia; Marlina@unsyiah.ac.id; 3 Computer Engineering Department, Faculty of Engineering, Bina Nusantara University, Jakarta 11480, Indonesia; rinda@binus.edu; 4 Department of Electrical Engineering, Krida Wacana Christian University, Jakarta 11470, Indonesia; indra.karnadi@ukrida.ac.id; 5 Research Center of Maju Makmur Mandiri Foundation, 40/80 Srengseng Raya, Jakarta 11630, Indonesia; kurnia18@cbn.net.id; 6 School of Industrial Technology, Universiti Sains Malaysia, Penang 11800, Malaysia; ngolaiya@futa.edu.ng; 7 Department of Physics, Faculty of Mathematics and Natural Sciences Universitas Syiah Kuala, Banda Aceh 23111, Indonesia

**Keywords:** castor oil, LIBS, adsorption, polyurethane, characterization

## Abstract

The use of polymeric material in heavy metal removal from wastewater is trending. Heavy metal removal from wastewater of the industrial process is of utmost importance in green/sustainable manufacturing. Production of absorbent materials from a natural source for industrial wastewater has been on the increase. In this research, polyurethane foam (PUF), an adsorbent used by industries to adsorb heavy metal from wastewater, was prepared from a renewable source. Castor oil-based polyurethane foam (COPUF) was produced and modified for improved adsorption performance using fillers, analyzed with laser-induced breakdown spectroscopy (LIBS). The fillers (zeolite, bentonite, and activated carbon) were added to the COPUF matrix allowing the modification on its surface morphology and charge. The materials were characterized using Fourier-transform infrared (FTIR), scanning electron microscopy (SEM), and thermal gravimetry analysis (TGA), while their adsorption performance was studied by comparing the LIBS spectra. The bentonite-modified COPUF (B/COPUF) gave the highest value of the normalized Pb I (405.7 nm) line intensity (2.3), followed by zeolite-modified COPUF (Z/COPUF) (1.9), and activated carbon-modified COPUF (AC/COPUF) (0.2), which indicates the adsorption performance of Pb^2+^ on the respective materials. The heavy metal ions’ adsorption on the B/COPUF dominantly resulted from the electrostatic attraction. This study demonstrated the potential use of B/COPUF in adsorption and LIBS quantitative analysis of aqueous heavy metal ions.

## 1. Introduction

Polymeric materials have been proposed for the removal of toxic heavy metal from environmental waste [1]. Heavy metal pollution is the release of toxic metals such as lead, chromium, cadmium, etc. into the environment. Major sources of these heavy metals are petroleum products, industrial waste, and soil leaching [1]. The ubiquity of heavy metal pollutants in the aqueous environment is caused by their release from the industrial wastewater. Heavy metal exposure on the human body can lead to serious multiple health damages, particularly heavy metals with a bioaccumulation ability such as Pb and Cr. Cr is infamous for its ability to act as a carcinogen and anaphylactogen in the human body. Meanwhile, Pb can cause damages to the circulatory system and control the nervous system. Therefore, the industries must be careful of the possibility of releasing these heavy metals to the environment after the wastewater management process.

Despite the eminence of adsorption reported by many studies [2,3], in practice, adsorption is always incorporated with the other wastewater purification methods such as reverse-osmosis filtration, electrocoagulation, and flocculation [4]. Thus, the development of adsorption, either the technique or the adsorbent materials, still gains its importance. The water released from these processes should have the heavy metal concentration below the threshold determined by government regulation. The information about the aqueous heavy metal concentration should be generated instantly to increase the efficiency of the whole wastewater management system.

Laser-induced breakdown spectroscopy (LIBS), is a newly developed analytical technique that can be utilized to perform instant qualitative and quantitative measurements for multi-elemental analysis [5,6]. Compared to other conventional techniques such as atomic absorption spectroscopy (AAS) or UV-VIS spectrophotometry, LIBS requires less sample treatment, thus offering more practicality and eco-friendliness. Even LIBS is more preferable than inductively coupled plasma atomic emission spectroscopy (ICP-AES) due to its applicability for in situ measurement. However, for aqueous samples, LIBS is still limited due to the liquid splashing and the water vaporization that contribute to the unreliable analysis [7]. To overcome the problem, adsorption has been incorporated to allow concentration enrichment on a solid surface, thus increasing the measurement stability [8]. Indeed, other techniques such as dual pulse laser and liquid jet have been developed to support LIBS analysis for aqueous samples [9], but those techniques lack practicalities and require complex equipment setups.

Polymeric materials have been extensively studied for their application in toxic metals removals, such as alginate-based composite [10], β-cyclodextrin nano-sponge [11], and cellulose membrane [12]. Other than those polymers, polyurethane (PU) has gained many interests in academic research for its adsorption ability to remove multiple heavy metal ions [13,14]. The interest in PU adsorbent is because the material can be easily manufactured and shaped [15]. Led by the rise of environmental concern, a new trend in PU research has been shifted to the use of bio-polyol resources. Lignin, starch, cellulose, chitosan, and vegetable oils (including castor oil) are some examples of many bio-polyols that have been used by researchers in PU synthesis for multiple applications in either pharmacy, building construction, packaging, agriculture, or other fields [16,17,18,19,20,21].

This study used castor oil as the bio-polyol feed because, unlike many other vegetable oils, castor oil possesses active secondary hydroxyl groups, which can react with the isocyanate to form a urethane linkage [15]. Due to its easy shape-tailoring, the addition of water into the polyol and diisocyanate mixture is enough to get the polyurethane foam (PUF) [22]. This material can appear in an open or closed cell structure with a wide range of rigidity (rigid, semirigid, and flexible foam) [19]. Hence, PUF has been noticeable for its high applicability as a thermal insulator [16], adhesive [17], and fertilizer release controller [21], and many others. In this research, however, we will employ PUF’s applicability in heavy metal removal by incorporating activated carbon, bentonite, and zeolite as modifying fillers. These fillers are prominent heavy metal adsorbents that have been reported by many studies [3,23,24,25,26].

The idea of this research was to develop an adsorbent material that can be used simultaneously in the wastewater management system and the heavy metal concentration monitoring with LIBS analysis. This article reports on the qualitative investigation of the Pb^2+^ adsorption behavior on the modified PUF using LIBS spectra. The characteristics of the newly developed materials and how they contribute to the adsorption performance are also reported in this article. It is important to figure out the adsorption behavior and how it contributes to a more sensitive and reliable quantitative analysis, especially with the contact time and solution acidity configurations.

## 2. Materials and Methods

### 2.1. Materials

The polyol source used in this research was commercial castor oil (*Ricinus communis* L.), purchased from Perseroan Terbatas (PT), Rudang Jaya, Kota Medan, Indonesia. Castor oil consists of approximately 90% ricinoleic and other fatty acids like linoleic acid (4%), oleic acid (1%), and linolenic acid (<1%). Bentonite and zeolite (mordenite) fillers used were natural clays, purchased from the local supplier Commanditaire vennootschap (CV), Agung Menara Abadi, Bandung, Indonesia. Meanwhile, other chemicals, methylene diphenyl diisocyanate (MDI), lead (II) acetate (Pb (CH_3_COO)_2_) as a feed for Pb^2+^, potassium chromate (K_2_Cr_2_O_7_) as a feed for Cr (VI), and activated carbon were all analytical grade and purchased from Sigma-Aldrich, St. Louis, MO, USA, except for the deionized water. 

### 2.2. Synthesis of Modified PUF

Polyurethane foam was synthesized with a simple one-shoot method, where the polyol mixture containing castor oil, glycerol, deionized water, and fillers was mixed with MDI. Firstly, polyol mixture was made by adding castor oil, glycerol, deionized water, and fillers (for modified COPUFs), mixed continuously at 750 rpm and 70 °C for 15 min (list of compositions can be seen in Table 1). After that, the MDI was added dropwise at the same rotation speed and temperature for another 20 s until the air bubbles were formed. The ratio of castor oil and MDI was 2:1, as adding more or less MDI than the stated ratio resulted in a deformed foam structure [22]. The modified COPUF with zeolite, bentonite, and activated carbon filters were respectively denoted as Z/COPUF, B/COPUF, and AC/COPUF. 

The procedure was followed by pouring the casting solution onto an aluminum mold 6 × 6 × 3 cm^3^ and left for around 10 s. After that, the casting solution was mixed with a glass rod by hand to avoid the accumulation of the fillers at the bottom of the mold and to prevent the void formation. The casting solution was cured in a vacuum oven at 70 °C for 3 h and then left for 24 h. Foam obtained from this process was cut into 1 × 2 × 0.5 cm^3^ and soaked in 100 mL acetone for 15 min. The foam pieces were removed from acetone and dried in an oven at 70 °C for 24 h. All foam pieces were stored in a zipped plastic bag for future uses.

### 2.3. Characterization

The functional groups and surface morphology of all synthesized PUFs were, respectively, characterized with Fourier-transform infrared (FT-IR) (Shimadzu FT-IR, Prestige 21 Series) and scanning electron microscopy (SEM) (Jeol, Jsm, 6510 LA). The thermal degradation profile was analyzed using thermal gravimetric analysis (TGA) (Shimadzu DTG-60) with nitrogen ambient (flow rate 20 mL/min) and a temperature rate of 10 °C/min.

Meanwhile, the swelling degree profile was constructed based on the water absorption into the foam. The foams were, respectively, added into a container filled with deionized water. The foams were removed, placed on a filter paper for 10 min, and weighed sequentially. The weighing was conducted every 24 h in 8 days. The swelling degree was calculated based on the difference of the foam weight before (*W*_0_) and after (*W_t_*) the soaking, divided by *W*_0_ (Equation (1)).

(1) $$\mathrm{Swelling}\;\mathrm{Degree}\;\left(\mathrm{SD}\right)=\frac{W_{t}-W_{0}}{W_{0}}$$

### 2.4. Batch Adsorption

Artificial Pb^2+^ and Cr (VI) stock solutions (1000 µg/mL) were prepared by dissolving Pb (CH_3_COOH)_2_ and K_2_Cr_2_O_7_, respectively. For adsorption studies, each stock solution was diluted to 100 µg/mL with deionized water. Then, 30 mL heavy metal solution 100 µg/mL was added into an Erlenmeyer 100 mL, and 0.2 g adsorbent was added to the Erlenmeyer and left on the shaker at 250 rpm. The adsorption was conducted with the variation of contact time (30, 60, 120, and 180 min) and pH (2, 4, and 7).

### 2.5. LIBS Analysis

After removal from the heavy metal solutions, the adsorbents were dried under the sunlight for around 3 h. The adsorption performances of the adsorbents were analyzed using LIBS. In the experiment, a Q-switched neodymium-doped yttrium aluminum garnet (ns Nd:YAG) laser (Quanta Ray, LAB 130-10, Spectra-Physics, Inc., Santa Clara, CA, USA) operating at 1064 nm with a 10 Hz repetition rate and energy of 83 mJ/pulse was employed to ablate the sample and to generate the plasma at an ambient air pressure of 760 Torr and 3 Torr. The laser beam was focused on the sample by using a quartz lens with a focal length of 150 mm. For low-pressure analysis (3 Torr), the sample was placed in a vacuum-tight metal chamber (11 cm × 11 cm × 12.5 cm). The plasma emission was collected using an optical fiber, which guided the light to the input slit of a spectrograph (Andor model 2061, focal length 1000 mm, f/8.6 Czerny Turner configuration). The exit slit of the spectrograph was equipped with a gated intensified charge couple devices (ICCD) (Andor Technology, iStar intensified CCD 1024 × 256 pixels, Belfast, UK). A digital delay generator (DG 535, Stanford Research System, Sunnyvale, CA, USA) was used to trigger the ICCD. The gate delay and gate width of the ICCD was fixed at 1 µs and 20 µs, respectively. Each spectrum was acquired from 10 shots of accumulation. During the experiment, the sample was mounted on a rotating holder to ensure a fresh surface for each laser shot. The sample holder was rotated at 5 rpm.

## 3. Results and Discussion

### 3.1. Reaction of Modified PUF and Functional Groups’ Analysis

**Figure 1:**
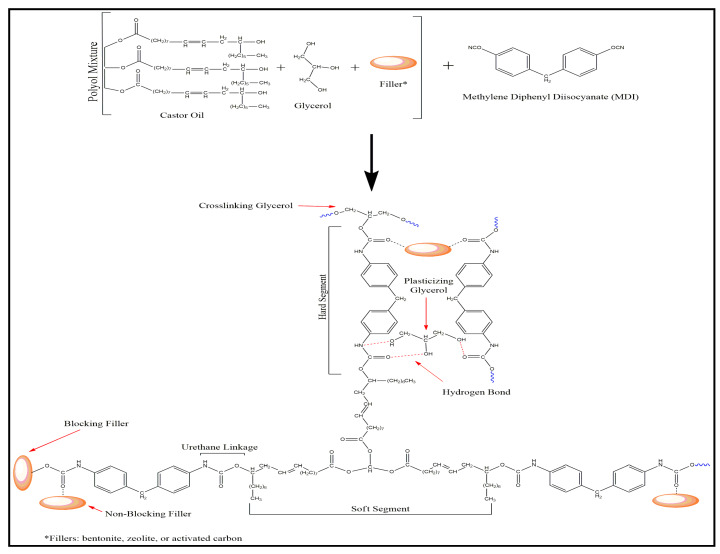
The reaction of castor oil-based PUF via the one-shoot method with the addition of glycerol and fillers.

The castor oil-based polyurethane foam (COPUF) was obtained via a one-shoot method, where the reaction and interaction with the modifying filters are shown in Figure 1. Ricinoleic acid, in the castor oil, contains a hydroxyl group that can be reacted to obtain a urethane linkage while other fatty acids can act as chain extenders [27]. MDI reacts with the secondary hydroxyl group (O–H) of castor oil triglyceride through a rearrangement reaction, forming a urethane linkage. The reaction was spontaneous without the help of a catalyst, due to the reactive nature of the MDI. The COPUF obtained was flexibly ascribed to the dominance of the long carbon chain from the castor oil, which contributes to the soft segment.

The addition of glycerol to the polyol mixture prevented the shrinkage and increased flexibility. Without the addition of glycerol, the foam experienced shrinkage and had higher rigidity [14]. Glycerol acted as a cross-linking agent, allowing the COPUF to sustain its structure, especially during the foam development phase. Besides, glycerol could also act as a plasticizer, where it interferes with the hydrogen bond and dipole-dipole interactions between the MDI chain [28]. The addition of the fillers also played a role in determining the physical structure of COPUF. 

**Figure 2:**
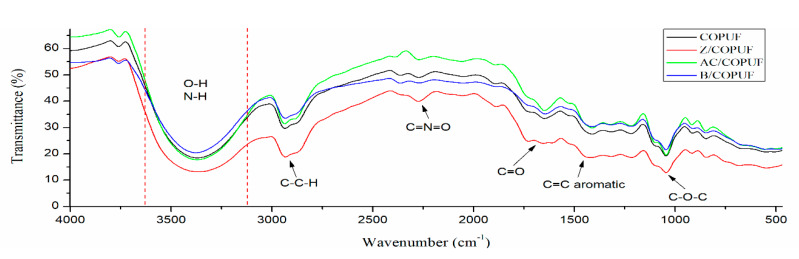
FTIR spectra of COPUF, Z/COPUF, AC/COPUF, and B/COPUF.

According to the FTIR analysis (Figure 2), the interaction between the fillers and COPUF was observed by the shifted wavenumber of C=O at around 1600 cm^−1^. It stemmed from the disturbance of filler particles on the electron vibration of the matrix, as reported previously [29]. In this case, the fillers may have blocked the polymerization by chemically interacting with the C-O from the MDI chain. The activated carbon (AC) was more likely to have this interaction due to the abundant hydroxyl groups it possesses. It explained the higher shrinkage on the AC/COPUF, compared to Z/COPUF or B/COPUF. The nonblocking fillers may have had weak intermolecular interaction, thus not affecting the propagation of the polymerization. 

Figure 2 also confirms the presence of N-H and O-H groups by a broad absorbance band at 3300–3500 cm^−1^, which is typical for polyurethane. The absorbance spectrum for C–C–H at around 2900 cm^−1^ and C=O at around 1600 cm^−1^ are associated with the typical functional groups of vegetable oil-based PUF. These functional group similarities may suggest the hydrophobic nature of COPUF, inherited from the castor oil.

Absorbance at the wavenumber 2200 cm^−1^ indicates the presence of C=N=O, which is associated with the excess of MDI. Z/COPUF had the highest absorbance intensity at the wavenumber (around 50% higher), indicating the unreacted MDI [14]. This is because the big size of zeolite particles may form an aggregate, thus hindering the reaction between the hydroxyl groups from the castor oil and the MDI’s isocyanate groups. Therefore, it is fair to conclude that the bentonite had a more homogenous distribution in the foam matrix than the distribution of the zeolite. The FTIR spectra also suggested the presence of C=O (1600 cm^−1^), aromatic C=C (1400 cm^−1^), and C–O–C (1200 cm^−1^). Those functional groups, along with N-H, are responsible for the heavy metal adsorption [23].

### 3.2. Scanning Electron Microscopy (SEM)

**Figure 3:**
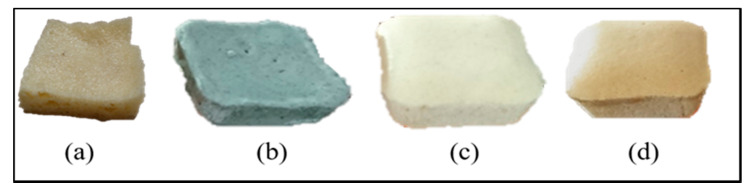
(**a**) COPUF, (**b**) AC/COPUF, (**c**) Z/COPUF, (**d**) B/COPUF.

The visual appearance of each foam is shown in Figure 3. It can be observed that the Z/COPUF and AC/COPUF had a lower volume compared to B/COPUF.

**Figure 4:**
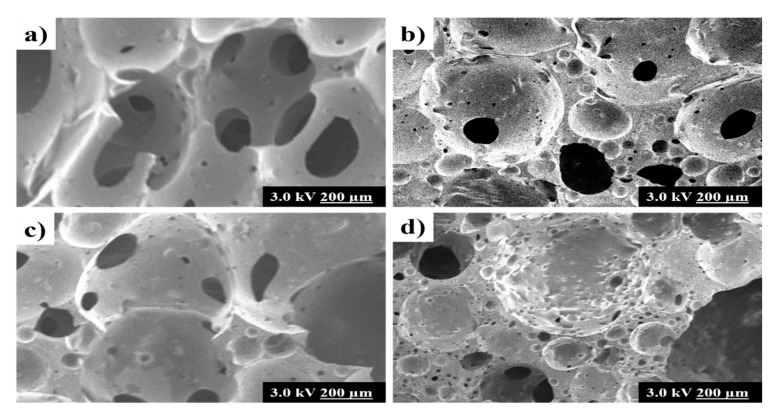
SEM images of (**a**) COPUF, (**b**) Z/COPUF, (**c**) B/COPUF, and (**d**) AC/COPUF.

Previous studies have reported the foam shrinkage after the addition of fillers due to the increase of foam density, which similarly happened in this research [23,30,31]. It is then confirmed by the relatively more closed pore structure in Z/COPUF and AC/COPUF from the images shown in Figure 4. As an addition, more pore opening was observed in B/COPUF compared to COPUF. The effect of fillers on the modification of the surface morphology is ascribed to their particle size and weight. As opposed to zeolite and activated carbon, bentonite appeared in finer particle size. This did not significantly affect the foam density. 

Other than the change in pores, the foam surface appeared to be rougher after fillers’ addition. The presence of filler particles, either bentonite, zeolite, or activated carbon, contributed to the formation of a rougher surface. Rough morphology facilitated more interaction with the adsorbent as it increased the contact surface [32]. It can be expected that the higher Pb^2+^ adsorption can be obtained from the foam modified with filler addition.

### 3.3. Swelling Degree Profile

**Figure 5:**
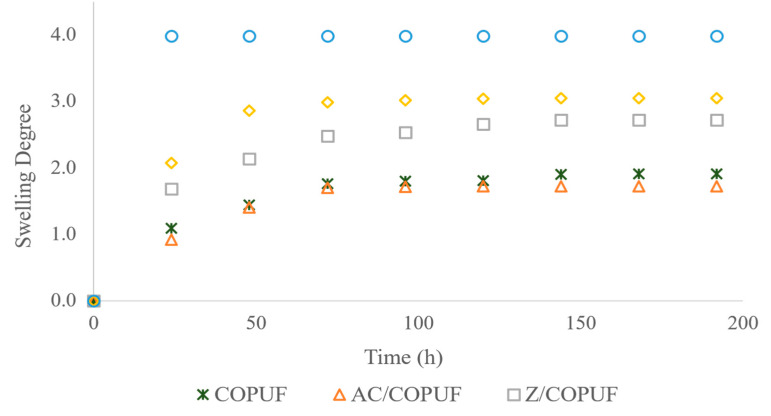
Swelling degree profile of COPUF, AC/COPUF, Z/COPUF, B/COPUF, and commercial PUF (CPUF).

Among the foams tested (COPUF, AC/COPUF, Z/COPUF, B/COPUF, and commercial PUF (CPUF)), the highest swelling degree value and the quickest equilibrium time were given by CPUF (Figure 5). The CPUF was labeled as super-hydrophilic by the manufacturer; hence, no wonder it performed with such superior water absorbance. Z/COPUF and B/COPUF came after CPUF, where both of them were modified with clay filler. The addition of clays was substantiated to increase the hydrophilicity of the material [33,34]. It was due to the charged clay surface, which facilitated more interaction with the water molecule. To increase the adsorption, hydrophilicity was expected to be increased, as it can assist the transportation of water molecules, which are the adsorbate carrier, to the inner foam structure. In contrary to the hydrophobic surface, which is typical for the vegetable oil-based polyurethane, it formed a barrier that obstructed the adsorbate transport.

Nevertheless, the high swelling degree sometimes is not expected as it can cause water absorption-induced deformation to the foam structure. High water absorption also means more time required to dry the sample, giving an inefficient adsorption-assisted LIBS application due to the increase in time consumption of the sample preparation. The combination of vegetable oil (hydrophobic) and clay (hydrophilic) can help to reach the proper hydrophobic–hydrophilic balance. Thus, the material obtained can have a good property to help the heavy metal adsorption, as well as good physical stability against water absorption.

### 3.4. Thermal Degradation Profile

**Figure 6:**
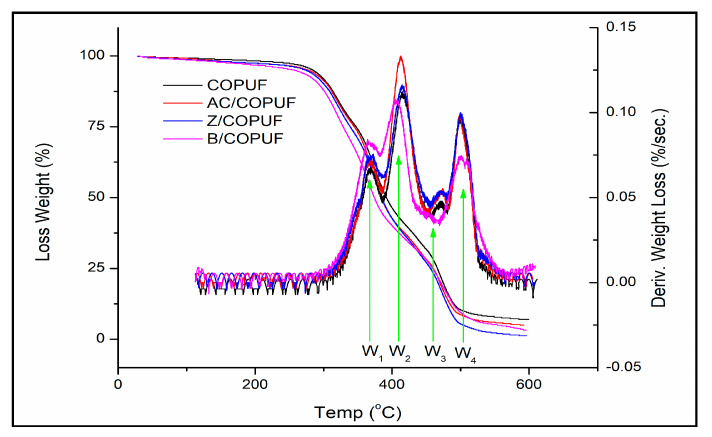
TGA and DTGA of COPUF, AC/COPUF, Z/COPUF, and B/COPUF with four stages of thermal degradation indicated by W_1–4_.

The thermal degradation profile of the COPUFs is presented with thermal gravimetry analysis (TGA) and its derivative (DTGA) curves, as can be observed in Figure 6. The thermal characteristic of PU can be identified through its typical four stages of degradation, as reported by Tenorio-Alfonso, Sánchez, and Franco (2019) [17]. The presence of a hard segment in the PU-urea system can be identified by the first two stages of thermal degradation (W_1–2_) at 270–414 °C, which are comparable with the previous reports [17,18,35]. The urea linkage originated from a further reaction of isocyanate and amine, resulting from isocyanate and water reaction [19]. The following two decomposition stages, at 414–533 °C, are respectively attributed to the castor oil chain scission (W_3_) and the breakdown of the C-C bonds (W_4_). 

The four COPUFs had a similar thermal characteristic, observed from the temperature of the indicated DTGA peaks, with T_onset_ at 241–252 °C and T_max_ at 363–373 °C. Slight differences of those temperature points may be ascribed to the reactive isocyanate leftover [17], as can also be seen in the FTIR analysis. Due to this effect of the excessive isocyanate, it was also difficult to determine and to compare the thermal stability of the final material. 

### 3.5. Adsorption of Pb^2+^ Ions on Various COPUFs

**Figure 7:**
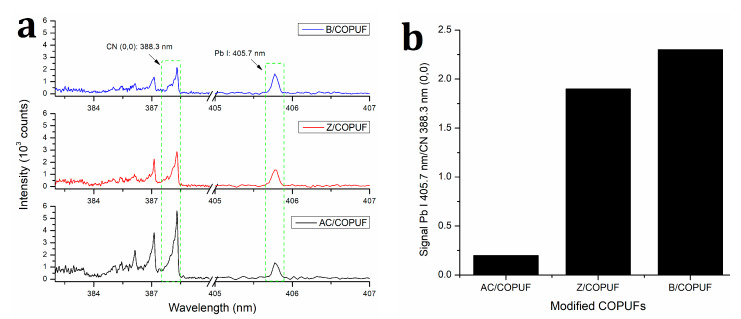
(**a**) LIBS spectra and (**b**) Pb I 405.7 nm/CN 388.3 nm (0,0) peak signal ratio of AC/COPUF, Z/COPUF, and B/COPUF after Pb^2+^ adsorption, analyzed under high air pressure (760 Torr).

Through the characterization results mentioned above, it is understood that the three materials synthesized in this research gave a different performance on Pb^2+^ adsorption. Therefore, to evaluate their respective performances, LIBS analysis was carried out by analyzing the difference of Pb I (405.7 nm) signal intensity given by each material. The use of LIBS in analyzing the adsorption ability of heavy metals on polymeric material is scarcely reported. Though Santos et al. used polymeric material (polyvinyl chloride), it was applied as a membrane [7]. Thus, this can be a significant contribution to analyzing the adsorption on COPUFs, especially with the presence of matrix effects such as roughness and uneven microsurface structure. To overcome the matrix effect, the sample was rotated at 5 rpm to get a new ablation spot, with 10 laser shot accumulations. The obtained LIBS spectra, which can be seen in Figure 7a, exhibited the presence of Pb I line at 405.7 nm from each modified castor oil-based polyurethane foam (AC/COPUF, Z/COPUF, and B/COPUF) after the adsorption of Pb^2+^ 1000 µg/mL was carried out. 

Due to the different properties of each material, including the porosity, thermal stability, water content, and, most importantly, the filler components, the normalization was a must. By using the emission line at 388.3 nm, from the CN band, like the standard line, each line intensity was plotted in a Pb I 405.7 nm/CN 388.3 nm (0,0) signal ratio graph (Figure 7b), which suggested higher Pb^2+^ adsorption on the B/COPUF than on the others. The selection of the standard line referred to the organic nature of COPUFs, in which the appearance of the CN band line is always consistent. Other potential standard lines, such as Ca I 422.6 nm that have close ionization energy with Pb I (405.7 nm), could not be used since the Ca content in each sample was different due to different modifying fillers added [36]. The same reason also applied to C I (247.8 nm), since COPUF with AC fillers had higher carbon content. 

These results are ascribed to the characteristics of B/COPUF, where it had more pores and higher hydrophilicity, allowing the easier transportation of the adsorbate (Pb^2+^) to the adsorbent surface. It can also be observed that Z/COPUF also gave a high Pb I line intensity as close as B/COPUF. This is because both modifying fillers (zeolite and bentonite) contributed to the higher hydrophilicity. COPUF is hydrophobic, stemming from the fact that the polyol monomer used to prepare the foam was hydrophobic vegetable oil. The addition of clay fillers, such as zeolite and bentonite, increased the hydrophilicity of the foam by contributing the charged active sites on the surfaces, allowing more interaction with the water. High hydrophilicity, however, may have contributed to the weaker water resistance; thus, it is also important to build a material with a good balance of hydrophilicity and hydrophobicity.

Activated carbon (AC) is known as one of the cheap adsorbents because it can be obtained from various bio-masses with high abundance. It has a unique structure, surface area, porosity, and functional groups, which contribute to its adsorption performance of heavy metals [23,24]. The addition of AC into the PUF matrix gave a result of rougher surface and higher water absorption, which was expected to be a good filler for facilitating higher Pb^2+^ adsorption [32]. Unfortunately, based on the spectral analysis in Figure 7a,b, COPUF modified with AC filler (AC/COPUF) gave the worst adsorption performance among the three modified foams. It is because the addition of AC caused foam shrinkage, leading to an increase in foam density and closing its pores, as corroborated by the SEM images (Figure 4). 

**Figure 8:**
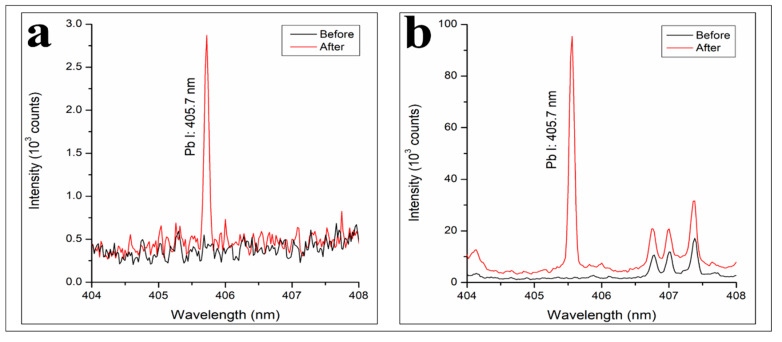
LIBS spectra of (**a**) COPUF and (**b**) B/COPUF before and after Pb^2+^ (100 µg/mL) adsorption, analyzed under low air pressure (3 Torr).

To understand whether the COPUF without modification could perform adsorption, the material was analyzed with LIBS after the adsorption of Pb^2+^ 100 µg/mL was carried out. Originally, the Pb I line (405.7 nm) was not observable when the same experimental conditions were used (760 Torr) before (Figure 6). Therefore, a chamber was used to change the experiment environment to have low ambient air pressure (3 Torr). Lowering the air pressure then gave a result that the Pb I line (405.7 nm) became observable, as shown in Figure 8.

Despite its hydrophobic behavior, the Pb^2+^ adsorption was found to be performed by the COPUF. Firstly, water transportation may have occurred due to the presence of small pores, allowing a capillary force to drive the water to reach the inner foam surface. This result was in the same agreement with the swelling degree profile (Figure 5) that, though slowly, the water absorption equilibrium was achieved after 144 h. 

Secondly, the presence of active groups (C=O and N-H), as indicated by the FTIR spectrum (Figure 2), may have contributed to the Pb^2+^ adsorption on the COPUF surface via chelation. As opposed to COPUF, modified foam AC/COPUF, Z/COPUF, and B/COPUF had more active sites to bind the Pb^2+^ on the surface. Hence, it gave more possible adsorption mechanisms, as shown by the high Pb I line intensity given by the B/COPUF, analyzed under the same air pressure (Figure 7a,b). Apart from its microporous structure, bentonite had a charged surface due to the substitute of its Al^3+^ cation, thus allowing the adsorption to occur with ion exchange and electrostatic force circumstances.

It is also important to notice the effect of air pressure, where it has been substantiated that lower air pressure gives higher Pb I intensity. Under air pressure 760 Torr, the generated Pb I line (405.7 nm) had an intensity of 1000 (a.u). The intensity was given by the B/COPUF after the adsorption of Pb^2+^ 1000 µg/mL. On contrary, under the low air pressure (3 Torr), even when the Pb^2+^ initial concentration was 10 times lower (100 µg/mL), the Pb I line intensity from B/COPUF was observed to be higher (88,900 (a.u.)). Even the unmodified foam (COPUF) that was understood to have a lower adsorption ability still gave higher Pb I line intensity (2400 (a.u.)). This result is comparable with Javed et al. (2018), where the optimum air pressure to observe Pb I line (405.7 nm) intensity was found to be around 3 Torr [6]. This air pressure was then used for the following experiment setup.

### 3.6. Effect of Contact Time 

**Figure 9:**
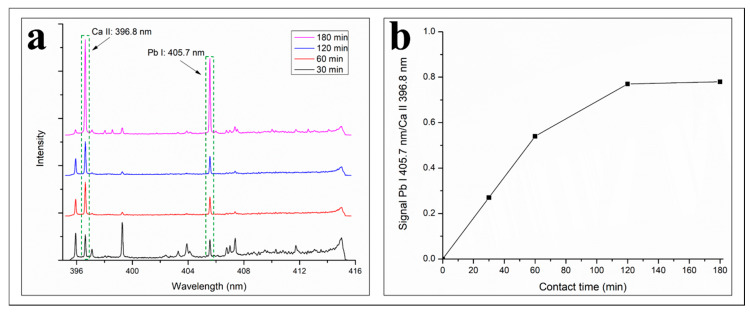
(**a**) LIBS spectra of B/COPUF after the adsorption of Pb^2+^ with a variation of contact time and (**b**) the Pb I/Ca II signal peak ratio.

Figure 9a exhibits the LIBS spectra of B/COPUF after Pb^2+^ adsorption with the variation of contact time, where each spectrum gave different signal peak intensity. The comparison between signal intensity cannot directly be made because of its dependence on the electron temperature. In order to compensate for the influence of electron temperature, the signal intensity of Pb I had to be divided with a standard line intensity. For this purpose, Ca (Ca II: 396.8 nm) was selected as the standard line because it is the only line with the highest intensity in the vicinity of the Pb I: 405.7 nm line.

The plot of Pb I 405.7 nm/Ca II 396.8 nm (0,0) signal versus contact time variation (30–180 min) can be seen in Figure 9b, where it suggests the increase of Pb^2+^ adsorption on B/COPUF along with the longer contact time. During 60 min of adsorption, the process was dominated by diffusion force. Afterward, the diffusion rate was slowing down as it almost reached the equilibrium. This phase was determined by the interaction between adsorbate and adsorbent surface. The adsorption reached its equilibrium after the 120th min, where a little to almost no change was observed in the 180th min.

For its application in the quantitative analysis of aqueous heavy metal ions, it is important to let the adsorption priorly reach the equilibrium point. The homogenous distribution of the adsorbed Pb^2+^ on the B/COPUF surface is less likely when the equilibrium is not reached, especially during the diffusion-dominant phase. It stems from the fact that some parts of the adsorbent surface may have been fully occupied by the heavy metal ions, while the other parts are still otherwise. Therefore, in the next experiment, the batch adsorption was run for 3 h to ensure the equilibrium was reached.

### 3.7. Effect of pH

**Figure 10:**
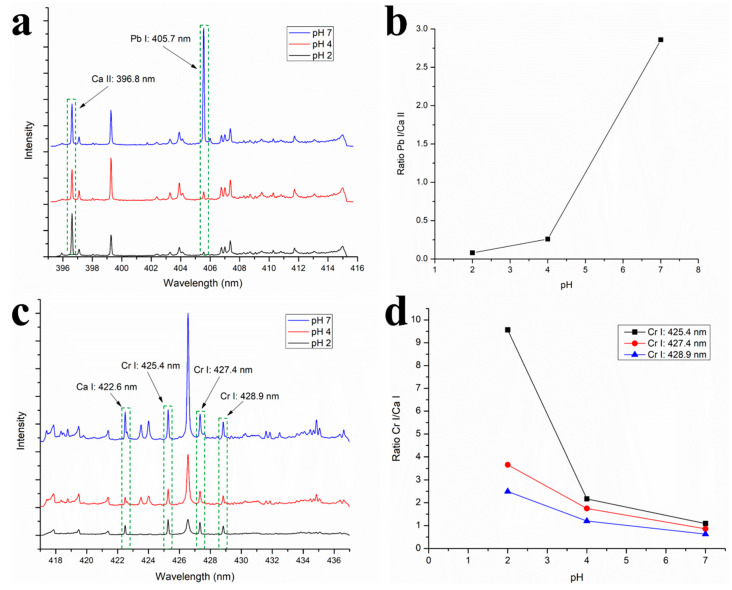
(**a**) LIBS spectra of B/COPUF and (**b**) the Pb I/Ca II signal peak ratio after the adsorption of Pb^2+^ (100 µg/mL) and (**c**) LIBS spectra of B/COPUF and (**d**) the Cr I/Ca I signal peak ratio after the adsorption of Cr (VI) (100 µg/mL).

As shown in Figure 10a,b, Pb^2+^ adsorption on the COPUF surface increased as the pH increased, indicated by the increase of intensity of the Pb I line at 405.7 nm. It was ascribed to the electrostatic force, ions competition, and the type of dominant ion species that play a major role in the adsorption. As reported by many studies previously, bentonite was observed to possess a negative charge in such pH. Charged surface attracts ion with the opposite charge. Thus, if the bentonite surface has a negative charge, it will attract Pb^2+^ ions. There are at least two reasons, why at low pH, Pb^2+^ adsorption is low: (1) The presence of H^+^ ions acting as the competitors and (2) the dominance of Pb^2+^ species. At higher pH, the presence of H^+^ decreased as the OH^−^ increased, resulting in less ion competition in the adsorption. At the same acidic level (pH 7), more Pb (OH)^−^ species were formed. In contrast with Pb^2+^ that occupied two active sites on the adsorbent surface, Pb (OH)^−^ only attracted to a single active site. Hence, more active site was left unoccupied.

However, the increase of pH reduced the adsorption Cr (VI), indicated by the change of the signal ratio of Cr I/Ca I (Figure 10c,d). Cr (VI) in the water appeared as an anion (Cr_2_O_7_^2−^) with a negative charge. When the pH was adjusted to 2, the system was reached with H^+^ ions. It then allowed the binding sites on the adsorbent surface to be occupied by H^+^, making the surface to be covered with a positively charged layer. It further attracted the negatively charged anion via a double electric layer [37]. Moreover, at low pH, Cr (VI) ion species were more dominated by HCr_2_O_7_^−^ [38], resulting in less of the required active site.

Note that, for Cr analysis, Ca I (422.6 nm) was selected as the standard line because Cr has similar ionization energy (Cr: 6.78 eV) [25]. Also, in this section of the analysis, each sample had the same proportion of Ca, since the same type and amount of filler (bentonite) was used. In a LIBS spectrum, Cr presence was indicated by the three signal lines, respectively appearing at 425.4, 427.4, and 428.9 nm. As reported by other studies, the signal line at 425.4 nm appeared to be the highest [25,38]. Nevertheless, the trend exhibited by Cr I/Ca I ratio at 425.4 nm was observably different from the other two. According to Wei et al. (2015), the Cr signal line at 425.4 nm was excluded from the quantitative analysis because its calibration curve gave the worst correlation [39]. Therefore, this paper focused more on observing the Cr signal line at 427.4 and 428.9 nm, as they show a better fit.

The effect of pH on Pb^2+^ and Cr (VI) adsorptions was a significant phenomenon because it suggested the possibility of a sample pretreatment option. By configuring the optimum adsorption pH, the Pb and Cr signals line could be intensified, thus allowing the detection and quantification of the heavy metal in small concentration. However, it is worth mentioning that the increase of pH more than 7 will result in precipitation. Heavy metal precipitation is an adverse effect on adsorption. Meanwhile, pH below 2 may destruct the adsorbent surface. Not only does it hinder adsorption performance [40], it also makes the adsorption-assisted LIBS quantitative analysis of the heavy metal unreliable because it does not follow the ideal adsorption. Therefore, the maximum observable pH effect in this study was at pH 7 for Pb and 2 for Cr.

The effect of contact time and pH on the adsorption of heavy metal on the polymeric surface was established in this study. Indeed, Rezk et al. (2018) had deeply studied the adsorption of Cu (II) and Co(II) on the fishbone adsorbent, but they did not use polymeric material [8]. Horňáčková et al. (2019) and Jiao et al. (2015) only slightly discussed the heavy metal adsorption without touching the effect of contact time and pH [25,39]. It is also worth mentioning that LIBS analysis opens possibilities of investigating heavy metal adsorption in such high concentration, which can give a better insight into the future adsorption studies.

### 3.8. Comparison with the Commercial Super-Hydrophilic Polyurethane Foam (CPUF)

**Figure 11:**
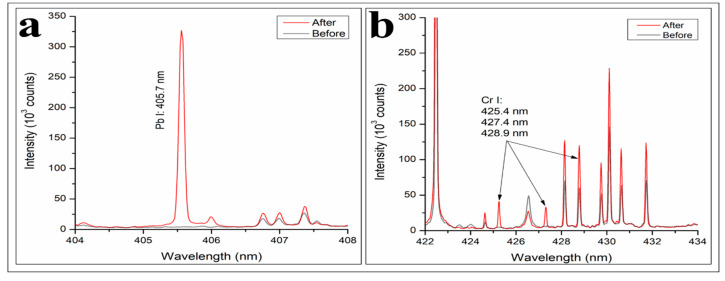
LIBS spectra of commercial PUF after and before the adsorption of (**a**) Pb^2+^ and (**b**) Cr (VI), analyzed at 83 mJ energy and 3 Torr air pressure.

According to the spectra presented in Figure 11a, commercial PUF (CPUF) gives a relatively higher Pb^2+^ adsorption compared to the PUFs prepared in this research. Even though the intensity of the Pb I line from CPUF and B/COPUF cannot be directly compared, the limit of detection (LOD) obtained from each LIBS spectrum can be used as the comparative parameter. The LOD values are calculated by Equation (2), as stated below:

(2) $$\mathrm{LOD}=\frac{3\left(\mathrm{Noise}\right)}{\mathrm{Signal}}\times \frac{10,000}{C}$$

where C is a concentration in µg/mL. The values of the detection limit obtained were 6 µg/mL and 1.9 µg/mL for B/COPUF and CPUF, respectively. It shows that CPUF had better Pb^2+^ adsorption than B/COPUF.

**Figure 12:**
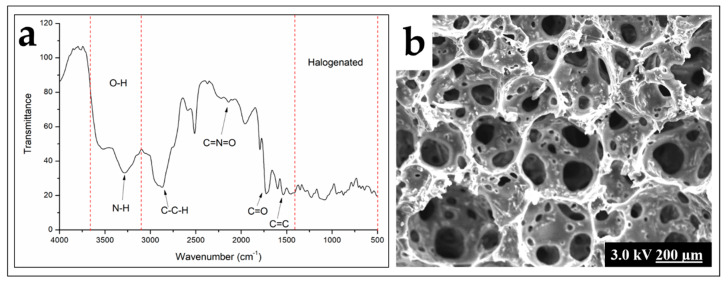
(**a**) Functional groups and (**b**) morphology characteristics of commercial PUF respectively analyzed with FTIR and SEM (200× magnification).

At least two significantly different characteristics distinguish the CPUF and B/COPUF, which can be observed from the FTIR and SEM analyses (Figure 12). Through the FTIR spectrum, besides the identical functional group it has with COPUF, CPUF is rich in alkyl halides (observed at around 1400–500 cm^−1^). These functional groups are responsible for allowing more interaction between the foam surface with Pb^2+^. The functional groups also lead to higher interaction between the foam surface and water, increasing its hydrophilicity. Under the same idea, smaller pore size, observed with SEM analysis, also facilitates a better heavy metal ions’ adsorption.

Figure 11b qualitatively showcases the ability of CPUF to adsorb Cr (VI), observed by the appearance of the Cr-typical three signal lines after the adsorption. Though the line at 428.9 nm had already appeared before the adsorption, the presence of Cr cannot be concluded, as there were not two other lines in the spectrum. Thus, the appearance of the three signal lines at 425.4, 427.4, and 428.9 nm confirm the ability of CPUF to adsorb Cr ions. Through the comparison of the LODs, B/COPUF performs a better Cr (VI) adsorption. The LODs of B/COPUF and CPUF respectively were 14 µg/mL and 20 µg/mL. It is because the binding sites of CPUF were mostly the alkyl halides (-F, -Cl, -Br, and -I), which possess high electronegativity. It then created an electrostatic repulsive barrier, hindering the adsorption of anionic Cr (VI). Hence, the adsorption of anions on the CPUF was less favorable than the adsorption of the cations.

Even though CPUF had the potential to assist LIBS qualitative measurement of heavy metals, B/COPUF was more preferable due to the environmentally friendly aspects. CPUF was made from a conventional polyol, which is a derivative product from fossil fuel separation. The manufacturing of fossil fuel derivative products is considered less eco-friendly and sustainable. In addition, the use of an abundantly available bio-polyol, such as castor oil, may reduce the cost of foam production. B/COPUF, which is prepared from the bio-polyol (castor oil), still has a lot of potentials, especially because it can perform better adsorption of Cr (VI). It suggests B/COPUF has effective adsorption of broad ionic heavy metal species. Therefore, in future research, we will improve the ability of B/COPUF in adsorbing the cations.

## 4. Conclusions

Castor oil-based polyurethane has been successfully manufactured and modified with the addition of activated carbon, zeolite, and bentonite as the fillers. The modification was confirmed through the character analysis with FTIR spectra (functional groups) and SEM images (surface morphology) of the respective modified COPUFs. The presence of typical functional groups of the castor oil explains the increased hydrophobicity of the material. The addition of zeolite and bentonite clay as the fillers were substantiated to improve its hydrophilicity, thus is more favorable for the aqueous heavy metal adsorption. SEM images confirmed that the filler addition changed the surface morphology, indicated by the roughness and the porosity.

The LIBS spectra comparison on the Pb I/CN ratio of COPUF, B/COPUF, Z/COPUF, and AC/COPUF after the adsorption, respectively, suggests that the highest adsorption occurred on B/COPUF. The adsorption of Pb^2+^ onto the adsorbent surface was driven by a diffusion force, which was largely affected by the hydrophilicity and the porosity of the foam. The equilibrium of Pb^2+^ adsorption was observed after 120 min contact time. The pH level also contributed to the different adsorption behaviors of the heavy metal ions. Where cationic Pb^2+^ gave strong adsorption at neutral-to-basic pH, anionic Cr (VI) adsorption preferred the H^+^-rich system.

B/COPUF had an advantage owing to its versatility that could accommodate the adsorption of both cations and anions. Meanwhile, the selectivity of the adsorbent could be adjusted by controlling the pH level. Compared to CPUF, B/COPUF had a better hydrophobic-hydrophilic balance, contributing to higher water-resistant property and faster drying process after the adsorption. Therefore, B/COPUF can be potentially applied and further developed to assist in LIBS quantitative measurement of aqueous heavy metals. Some adsorbent properties which can be focused on future improvement include but are not limited to hydrophilicity, pore size, and functional groups. 

## Figures and Tables

**Table 1:** Composition variations of castor oil-based polyurethane foams (COPUFs).

Sample	Filler	Castor Oil (g)	MDI (g)	Glycerol (g)	Deionized Water (g)
COPUF	-	10	5	4	2
Z/COPUF	Zeolite (0.1 g)	10	5	4	2
B/COPUF	Bentonite (0.1 g)	10	5	4	2
AC/COPUF	Activated Carbon (0.1 g)	10	5	4	2
